# Disease-modifying therapies for Rett syndrome: a review for neurologists

**DOI:** 10.3389/fneur.2026.1766679

**Published:** 2026-01-20

**Authors:** Debopam Samanta

**Affiliations:** Division of Child Neurology, Department of Pediatrics, University of Arkansas for Medical Sciences, Little Rock, AR, United States

**Keywords:** AAV, gene therapy, neurodevelopmental disorders, pediatric neurology, precision therapeutics

## Abstract

Rett syndrome (RTT) is a severe X-linked neurodevelopmental disorder affecting approximately 1 in 10,000–15,000 females, most often caused by loss-of-function mutations in *MECP2*. Until the recent approval of trofinetide, management relied exclusively on symptomatic treatment and multidisciplinary supportive care. The therapeutic landscape is now undergoing a rapid shift, driven by multiple gene therapy approaches designed to restore functional MeCP2 expression and achieve true disease modification. As these therapies progress toward potential regulatory approval, neurologists will play central roles in identifying eligible patients, counseling families, supporting clinical trial enrollment, delivering treatments, monitoring long-term outcomes, and advocating for equitable access. This review provides neurologists with the essential framework needed to understand and navigate this evolving field. We examine in detail the two most advanced gene replacement therapies currently in clinical trials. TSHA-102 uses an intrathecally delivered miniMECP2 transgene regulated by a microRNA-based autoregulatory system, whereas NGN-401 delivers full-length *MECP2* via intracerebroventricular administration using a synthetic expression-feedback circuit. Both approaches have shown encouraging early efficacy, with treated children achieving developmental gains that exceed natural history expectations. However, they differ substantially in molecular design, regulatory control, delivery method, and safety considerations. We also highlight challenges unique to RTT gene therapy, including the narrow therapeutic window between insufficient expression and MeCP2 overexpression toxicity, the impact of X-chromosome inactivation mosaicism, and lessons learned from a fatal hyperinflammatory adverse event. Beyond AAV-mediated gene replacement, we review next-generation strategies in preclinical development—CRISPR-Cas9 genome editing for permanent mutation correction, ADAR-based RNA editing, translation readthrough for nonsense variants, and X-chromosome reactivation to restore endogenous *MECP2* expression. Finally, we address key translational considerations such as optimal timing of intervention, dosing constraints, outcome measurement in severely impaired populations, long-term safety surveillance, and barriers to broad and equitable access. The RTT gene therapy experience serves as a model for precision medicine in other monogenic neurodevelopmental disorders, illustrating both the transformative promise and the substantial complexities of translating genetic science into meaningful clinical benefit.

## 1 Introduction

Rare diseases collectively affect a surprisingly large segment of the population, with an estimated 3.5–5.9% of individuals worldwide living with one of more than 6,000 recognized conditions—most of which are genetic and begin in childhood (1). Rare neurodevelopmental disorders represent a substantial portion of this burden and, although individually uncommon, they represent a major component of pediatric and adult neurology practice. Neurologists therefore serve as the primary long-term physicians for many of these patients, guiding diagnostic workup, symptom management, and multidisciplinary coordination across the lifespan. At the same time, the therapeutic landscape is rapidly evolving, with cell and gene therapies poised for unprecedented expansion—projected to exceed 200 FDA approvals and result in more than 100,000 patients in the United States receiving these treatments by 2030 (2). Despite this rapid acceleration, many neurologists remain unfamiliar with the genetic testing and patient-selection considerations of emerging disease-modifying therapies (3, 4). Rett syndrome (RTT)—one of the most recognizable monogenic neurodevelopmental disorders—offers a timely and clinically relevant example to illustrate how gene-directed approaches are reshaping expectations for treatment in neurology. This severe X-linked dominant neurodevelopmental disorder, affecting approximately 1 in 10,000–15,000 females, follows a characteristic and devastating trajectory: after a period of apparently normal early development, affected girls experience profound regression of motor and communication skills between 6 and 18 months of age, followed by stabilization with persistent severe impairment (5–8). The disorder is caused by loss-of-function mutations in the *MECP2* gene located on chromosome Xq28, which encodes methyl-CpG-binding protein 2 (MeCP2)—a transcriptional regulator essential for neuronal maturation, synaptic plasticity, and the maintenance of mature neuronal function (9, 10). Multiple sophisticated gene therapy strategies for RTT are now in advanced clinical trials, with several more in preclinical and discovery phases, yet a focused review for neurologists is lacking. This article provides a comprehensive, clinician-oriented overview of these emerging therapies, their scientific rationale, current clinical trial data, and the key challenges that remain.

## 2 Clinical features, natural history, diagnostic criteria, surveillance, and symptomatic management

Understanding the natural history of RTT is essential for interpreting therapeutic trial outcomes and counseling families about realistic expectations. The disorder follows a characteristic four-stage progression, though individual variability is substantial and staging may overlap (11) (Table 1). Diagnosis requires clinical criteria supported by molecular confirmation of a pathogenic *MECP2* variant (Table 2). RTT is associated with multiple comorbidities, including epilepsy, which affects 70–90% of individuals and is a major contributor to morbidity. Seizure types are diverse—focal seizures, generalized tonic–clonic seizures, atypical absence seizures, and myoclonic seizures are all reported (12). Up to one third of patients have drug-resistant epilepsy, which caregivers consistently identify as one of the greatest barriers to quality of life (13–17). Beyond epilepsy, additional comorbidities are common and require ongoing multidisciplinary care. Prior to the approval of trofinetide, no disease-specific therapy existed, and management focused primarily on surveillance and symptomatic treatment (18, 19) (Table 3). However, a major limitation—and a source of considerable frustration—is that none of these interventions are truly disease-specific or disease-modifying (20).

**Table 1:** Stage-specific clinical features in Rett syndrome.

Stage	Age range	Key features	Neurologic/Motor features	Behavioral features	Other features
I. Early Onset/Stagnation	6–18 months	Subtle developmental slowing	Hypotonia; decreased eye contact	Reduced exploration	Deceleration of head growth
II. Rapid Destructive	1–4 years	Rapid regression of skills	Loss of purposeful hand use; stereotypic hand movements; loss of language; gait apraxia/ataxia	Irritability; inconsolable crying; social withdrawal	Irregular breathing patterns; onset of seizures
III. Plateau/Pseudostationary	2–10 + years	Stabilization of regression	Persistent apraxia, ataxia; worsening scoliosis	Improved eye gaze and nonverbal communication	Growth retardation; peak seizure burden
IV. Late Motor Deterioration	Late childhood to adulthood	Progressive motor decline	Loss of ambulation; increasing rigidity, spasticity, dystonia	Behavioral improvement; fewer seizures	Increased risk of fractures and immobility complications

**Table 2:** Diagnostic criteria for typical and atypical Rett syndrome.

Category	Criteria
Main Criteria (all required for typical RTT)	Loss of purposeful hand skills Loss of spoken language Gait abnormalities (ataxia, apraxia, or loss of ambulation) Stereotypic hand movements
Supportive Criteria (≥5 required for atypical RTT)	Breathing irregularities while awake Bruxism Sleep disturbances Abnormal muscle tone Peripheral vasomotor disturbances Scoliosis/kyphosis Growth retardation Small, cold hands and feet Inappropriate laughing or screaming spells Diminished pain response Intense eye communication
Required for molecular confirmation	Documented pathogenic MECP2 variant

**Table 3:** Recommended evaluations after MECP2 diagnosis (with management considerations).

System/concern	Recommended evaluation	Comments	Management
Constitutional	Height, weight, head circumference	Baseline growth and nutritional status	Nutrition optimization; monitor for growth failure
Neurologic	Neurology exam, brain MRI; EEG/video EEG if seizures suspected	High seizure prevalence; evaluate tone/movement	ASM management; seizure safety education
Development	Developmental, motor, cognitive, speech-language evaluation	Determines therapy needs	Early intervention (0–3 yr), special education (3 + yr), PT/OT/SLP
Psychiatric/Behavioral	Neuropsychiatric screening (>12 months): sleep, ADHD, anxiety, ASD traits	Behavioral features common	Melatonin for sleep; SSRIs/risperidone for agitation/anxiety
Musculoskeletal	Ortho/PM&R; PT/OT assessment; scoliosis screening	High risk of scoliosis and mobility decline	PT/OT, bracing, positioning devices; surgical referral if needed
Gastrointestinal/Feeding	GI/nutrition evaluation; aspiration risk assessment	Constipation, reflux, feeding difficulty common	Constipation regimen; GERD therapy; feeding therapy; consider G-tube
Respiratory	Overnight sleep study	Evaluates apnea, hypoventilation	Respiratory support as needed; sleep interventions
Sleep disorders	Portable polygraphic screening	Detects apnea/hypopnea	Melatonin ± hydroxyzine/diphenhydramine
Cardiovascular	Cardiology evaluation, ECG	Risk of prolonged QTc	Avoid QT-prolonging drugs; cardiology follow-up
Osteopenia	Bone densitometry	High fracture risk	Optimize Ca/Vit D; encourage weight-bearing; follow bone health guidelines
Vision	Ophthalmology evaluation	Strabismus, visual impairment common	Vision therapy, school-based services
Hearing	Audiology evaluation	Rule out hearing loss	Hearing aids; educational supports
Genitourinary	Clinical evaluation	Functional incontinence common	Urology/pelvic floor referral as needed
Integument/Autonomic	Skin/vascular exam	Cold, poorly perfused hands/feet	Warmth strategies; monitor autonomic symptoms
Genetics	Geneticist/genetic counselor consult	Family counseling	Ongoing counseling; recurrence-risk discussion
Family resources	Social work; community support needs	High caregiver burden	Respite, home nursing, palliative care when appropriate

## 3 Trofinetide

Trofinetide, the first FDA-approved therapy for RTT, is a synthetic IGF-1–derived peptide designed to support synaptic function in MECP2-deficient neurons (21–23). Its approval followed a Phase 3 trial demonstrating modest but meaningful clinical improvement, with gastrointestinal side effects as the main tolerability concern (24–28). Open-label extension studies have shown consistent efficacy and no new safety signals (29–32) (Table 4). While trofinetide marks an important milestone and offers symptomatic benefit for some individuals, it requires chronic twice-daily dosing and does not address the underlying MECP2 deficiency—highlighting the need for more transformative, disease-modifying approaches.

**Table 4:** Summary of trofinetide clinical experience in Rett syndrome.

Study (Name/publication)	*N*/Age (yrs)	Dose/Regimen	Duration	Efficacy/Key findings	Adverse effects/Safety
LAVENDER (Phase 3 RCT), Neul et al. (25, 26)	187 (93 trofinetide/94 placebo); age 5–20 yrs.	Weight-based trofinetide, twice daily (oral)	12 weeks	Co-primary endpoints: RSBQ change −4.9 vs. − 1.7 (*p* = 0.0175), CGI-I 3.5 vs. 3.8 (*p* = 0.0030). Key secondary: communication/social (CSBS-DP-IT Social) improved (LS mean difference 1.0, *p* = 0.0064).	Diarrhea 80.6% vs. 19.1% placebo (mostly mild–moderate), vomiting 26.9% vs. 9.6% placebo. 17.2% discontinuation due to TEAEs in trofinetide arm vs. 2.1% placebo. Serious AEs 3.2% in both groups.
DAFFODIL (Phase 2/3 open-label) — Percy et al. (29)	15 girls; age 2–4 yrs.	Weight-based trofinetide, bid (oral)	Period A 12 weeks + longer-term follow-up (~21 months)	Exploratory efficacy: improvements in CGI-I, caregiver-reported global impression (CaGI-I), and quality of life (ICND-QoL). Caregiver exit interviews (*n* = 7) — all “satisfied” or “very satisfied.”	Treatment-emergent AEs: diarrhea 80%, vomiting 53.3%, mostly mild/moderate. PK data: weight-based dosing achieved target exposure.
LILAC (open-label extension of LAVENDER) — 2024 report	154 females; age 5–21 yrs.	Trofinetide, bid, open-label (oral)	40 weeks	RSBQ mean change from LAVENDER baseline to week 40: −7.3 (SE 1.62) in those originally on trofinetide, −7.0 (SE 1.61) in those switched from placebo. CGI-I at week 40 (from LILAC baseline): 3.1 (SE 0.11) for prior trofinetide, 3.2 (SE 0.14) for prior placebo.	AEs: diarrhea 74.7%, vomiting 28.6%, COVID-19 11%. Diarrhea caused withdrawal in 21.4%. No new safety signals beyond those in LAVENDER.
LILAC-2 (32-month open-label extension) — Percy et al. (29, 30)	77 females; age 5–22 yrs. (mean ~12.0 ± 4.4 yr)	Trofinetide, bid, open-label (oral)	Up to 32 months (median exposure ~811 days)	Sustained improvement: RSBQ change from LAVENDER baseline to week 104 of LILAC-2: −11.8 (SE 2.45). CGI-I at week 12 of LILAC-2 (from LILAC baseline): 3.1 (SE 0.10). Most caregivers (96%) reported being “satisfied” or “very satisfied” with treatment benefits.	AEs: diarrhea 53.2%, vomiting 19.5%, COVID-19 27.3%. No new safety signals.
LOTUS (real-world, observational, up to 12 months) — interim data 2025	227 patients (US, pediatric & adult), on trofinetide under routine care; age ≥2 yrs. (per approval criteria)	Weight-based, bid, oral or via gastrostomy tube per real-world prescription	Up to 12 months (interim)	Median dose at week 1: 36% of target, increased to >80% by week 10; 55.2% reached full recommended daily dose Caregiver-reported behavioral improvements (BIQ) months 1–12: Non-verbal communication 48–71%, Alertness 44–70%, Social interaction & connectedness 33–58% Median QI-Disability total score change from baseline: 4.7 → 4.6 (months 1–12)	Diarrhea: weeks 1–12 (23–50%), months 4–12 (26–38%), mostly contained in diapers Safety consistent with prior trofinetide trials

With the growing interest in rare disease–specific therapies, numerous pharmacologic treatments for RTT are currently in clinical and preclinical development (summarized in Table 5). However, this review specifically focuses on current and emerging gene therapy approaches.

**Table 5:** Rett syndrome pharmacological therapeutics in clinical and preclinical development (Excluding gene therapies).

Product name	Proposed mechanism of action	Current status in research
2–73 (blarcamesine)	Sigma-1 receptor activator	Phase 2/3 study in girls (5–17 years) completed in Australia, Canada, UK - did not meet statistical significance on primary endpoints at 12 weeks. Phase 3 adult study completed in Australia/UK with topline results expected soon. Has FDA Fast Track, Rare Pediatric Disease, and Orphan Drug designations.
Fenfluramine	Serotonin 5-HT2 receptor agonist	Phase 3 clinical program planned by UCB Pharma, expected to begin first half of 2026. Already FDA-approved for Dravet and Lennox–Gastaut syndromes.
Leriglitazone	Selective PPAR-*γ* agonist with CNS penetration; improves mitochondrial function, reduces oxidative stress and inflammation	Phase 2a TREE Study initiated March 2025 in Spain. Placebo-controlled, 36-week study in 24 pediatric patients (up to 17 years) with confirmed MECP2 mutations.
NTI164	Broad-spectrum medical cannabinoid	Phase I/II trial in Australia. After 12 weeks (May 2024), 93% of participants showed improvement on CGI-I scale with 205% mean improvement on RSBQ from weeks 4–12. All participants enrolled in 52-week extension. Has FDA Orphan Drug Designation (Nov 2024), EMA Orphan Drug Designation (March 2025), and FDA Rare Pediatric Disease Designation (Oct 2025).
Vorinostat (RVL-001)	Small molecule oral therapeutic (mechanism specific to Rett not detailed)	Clinical trial material manufacturing initiated in 2024 for trials in US and Colombia. Proof-of-concept “n-of-1” placebo-controlled trial planned for early summer 2025 in Colombia with 15 patients. Has FDA Orphan Drug Designation (May 2024).
DPM-1003	Protein tyrosine phosphatase (PTP) family enzyme inhibitor	FDA clearance received March 2024 to initiate Phase 1 clinical trial. Has Orphan Drug designation and conditional Rare Pediatric Disease designation.
MB-204	Adenosine A2A receptor antagonist; modified version of istradefylline (FDA-approved for Parkinson’s)	Preclinical stage. Mouse study data released March–April 2025 showed greater improvement in social behaviors vs. trofinetide. Plans to file for FDA Orphan Drug Designation announced March 2025.
NQ-13	Peptide-based IGFBP2 mimetic; involved in neurogenesis, synaptic function, and cognition	Preclinical development. In rat model, reversed movement, breathing, and cognitive dysfunction, and restored brain MeCP2 levels to normal values.
AMO-04	Glutamate modulator	Preclinical development. Granted FDA Orphan Drug Designation in June 2018. Showed promise in mouse model through IRSF’s Scout Program.
KIT-13/KIT-14	Synthetic plasmalogen derivative; anti-inflammatory effects via p65 nuclear accumulation inhibition	Preclinical development. KIT-13 granted FDA Rare Pediatric Disease and Orphan Drug Designations in March 2023. KIT-14 received EMA Orphan Drug Designation in June 2025.
NLX-101	Serotonin 5-HT1A receptor activator	Preclinical development. Published study in March 2025 showed improvements in respiratory impairment and cognitive deficits in Rett mouse model. Reduced apneas and normalized irregular breathing patterns.
OV4041	Direct KCC2 activator; restores excitatory/inhibitory balance and supports GABA inhibition	Preclinical stage. Plans announced June 2025 to begin preclinical studies in late 2025 for IND filing, with IND submission expected late 2026. Has anxiolytic, antipsychotic, and anticonvulsant activity.
Axonis Therapeutics (TBD)	KCC2-targeting medicines to maintain inhibitory signaling in the brain	Preclinical research. Collaborative grant with Tang Lab at Boston Children’s Hospital. Research showed increasing KCC2 expression can rescue cellular and behavioral abnormalities in Rett.
Pridopidine	Sigma-1 receptor (S1R) agonist with neuroprotective properties	Preclinical evaluation ongoing by Prilenia Therapeutics. Currently in late-stage development for Huntington’s disease and ALS.
Gliachem compounds	TRPM2 channel blockers; boost mTOR activity to restore brain development and function	Preclinical development. New molecules designed to block TRPM2, which negatively regulates mTOR and is over-expressed in Rett brain.
Palena Therapeutics EERPs	Immunomodulatory peptides (embedded epitope random peptides) that increase BDNF expression	Preclinical concept stage. Similar mechanism to Copaxone in MS. Small open-label study of Copaxone (n = 10) showed improvements in gait, visual attention, memory, and breath holding index.

PPAR-γ (Peroxisome Proliferator-Activated Receptor Gamma), CNS (Central Nervous System), CGI-I (Clinical Global Impression – Improvement), RSBQ (Rett Syndrome Behavior Questionnaire), FDA (Food and Drug Administration), EMA (European Medicines Agency), IGFBP2 (Insulin-Like Growth Factor Binding Protein 2), PTP (Protein Tyrosine Phosphatase), KCC2 (Potassium-Chloride Cotransporter 2), GABA (Gamma-Aminobutyric Acid), IND (Investigational New Drug), S1R (Sigma-1 Receptor), TRPM2 (Transient Receptor Potential Melastatin 2), mTOR (Mammalian Target of Rapamycin), EERPs (Embedded Epitope Random Peptides), BDNF (Brain-Derived Neurotrophic Factor), MS (Multiple Sclerosis), ALS (Amyotrophic Lateral Sclerosis), IRSF (International Rett Syndrome Foundation).

## 4 Gene therapies

Since the landmark discovery that restoring MeCP2 expression can reverse severe neurological symptoms — even in mature mice — there has been sustained optimism that gene therapy could meaningfully treat RTT (33). Especially, delivering a functional MECP2 gene to affected brain cells has long been considered a promising therapeutic strategy (34, 35). However, translating this preclinical promise to clinical reality faces a formidable challenge unique to RTT: the biology of X-chromosome inactivation. In females, one X chromosome is randomly inactivated in each cell during early development (lyonization), creating a mosaic pattern where approximately half of neurons express the mutant *MECP2* allele while the other half express the wild-type copy (36–40). Conventional gene replacement strategies risk delivering *MECP2* to cells already expressing the functional allele, potentially causing overexpression toxicity that mimics MECP2 duplication syndrome—a distinct disorder characterized by intellectual disability, autism, and epilepsy (41). Conversely, insufficient expression in *MECP2*-deficient neurons fails to achieve therapeutic benefit (42).

Gene replacement therapy for RTT has progressed slowly in mouse models because of multiple challenges: lentiviral vectors provide only limited distribution around the injection site; different generations of expression cassettes using various promoters have carried risks of systemic toxicity—particularly to the liver; transduction efficiency has often been insufficient to rescue phenotypes; newer capsid variants aim to improve blood–brain barrier permeability but may require immunosuppression; and although self-complementary AAVs offer higher efficiency, their reduced packaging capacity (≈2.2 kb) requires careful selection of essential transgene elements (43–50). Consequently, effective gene therapy for RTT demands sophisticated regulatory systems that enable self-tuning, context-dependent MECP2 expression—restoring levels in deficient cells while avoiding toxicity in wild-type cells. The most advanced strategies that have reached clinical trials are discussed below.

### 4.1 Gene replacement therapies: TSHA-102 and NGN-401

Two gene therapy candidates employing distinct regulatory strategies have entered clinical trials: TSHA-102 (Taysha Gene Therapies) and NGN-401 (Neurogene Inc.) (51). Both use AAV9 vectors for CNS delivery but differ fundamentally in their *MECP2* construct design, regulatory mechanisms, and administration routes.

#### 4.1.1 TSHA-102: miniMECP2 with miRARE technology

##### 4.1.1.1 Molecular design and preclinical foundation

**Figure 1:**
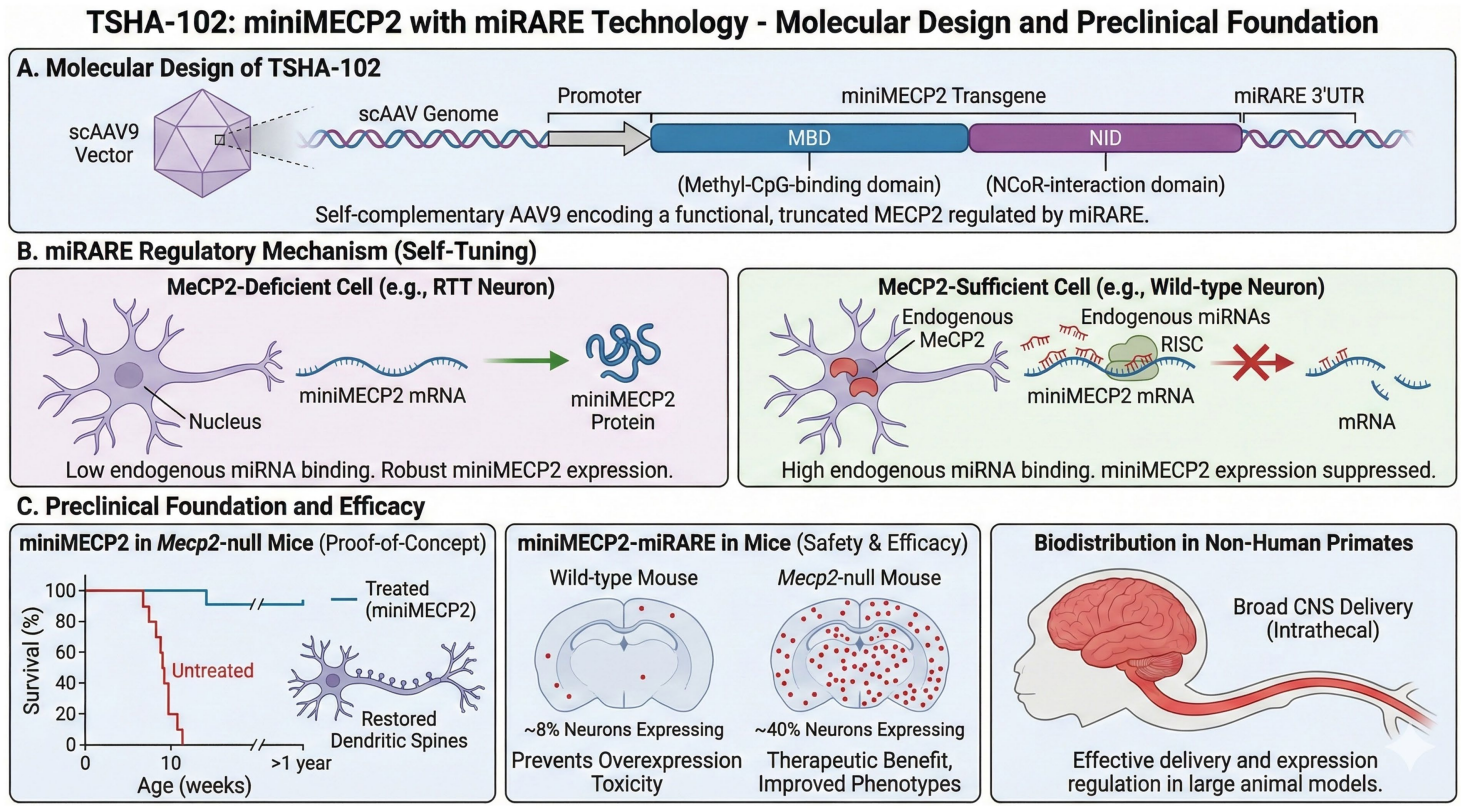
TSHA-102 molecular design, miRARE regulation, and preclinical efficacy. **(A)** Molecular design: TSHA-102 delivers a truncated “miniMECP2” via a self-complementary AAV9 vector, retaining the essential MBD and NID domains to fit the ~2.2 kb scAAV cargo limit. **(B)** miRARE regulatory mechanism: The miRARE platform inserts microRNA-responsive sequences in the 3’UTR, creating a negative feedback loop: cells with sufficient MeCP2 suppress transgene expression, while MeCP2-deficient cells allow robust expression. **(C)** Preclinical efficacy: In MECP2-null mice, scAAV-miniMECP2 improved neurological function, breathing, and survival without overexpression toxicity. In non-human primates, lumbar intrathecal delivery achieved broad CNS distribution.

TSHA-102 employs a self-complementary AAV9 (scAAV9) vector encoding a truncated “miniMECP2” transgene regulated by the proprietary miRARE (microRNA-Responsive Autoregulatory Element) platform (Figure 1). The miniMECP2 construct addresses a fundamental packaging constraint: scAAV vectors permit faster, more robust gene expression by forming double-stranded DNA without requiring cellular DNA synthesis, but they have a limited cargo capacity of only ~2.2 kilobases. Because the full-length MECP2 coding sequence exceeds this limit, a minimal functional version was engineered that retains only the essential domains: the methyl-CpG-binding domain (MBD), which recognizes methylated DNA, and the NCoR-interaction domain (NID), which recruits transcriptional repressor complexes (52). By delivering the essential MBD and NID functional domains, TSHA-102 provides a genotype-agnostic approach that encompasses the molecular defects found in over 95% of the RTT population, including the eight most common recurrent mutations and nearly all pathogenic missense variants.

Preclinical validation of miniMECP2 was conducted by delivering scAAV-miniMECP2 directly into the brains of neonatal Mecp2-null mice (52, 53). Treated animals demonstrated significant improvements in neurological function, normalized breathing patterns, and extended survival without the toxicity associated with full-length *MECP2* overexpression in wild-type mice (52). This established proof-of-concept that a truncated *MECP2* protein, if efficiently expressed, could rescue core RTT phenotypes.

However, even miniMECP2 poses risks of overexpression. To address this, the miRARE regulatory system was utilized (54). miRARE leverages endogenous microRNAs to create a negative feedback loop: multiple tandem sequences complementary to specific endogenous miRNAs are inserted into the 3′ untranslated region (3’UTR) of the miniMECP2 transgene. In cells where these cognate miRNAs are abundant (including neurons that already express sufficient MeCP2), miRNA binding recruits the RNA-induced silencing complex (RISC), which degrades the transgene mRNA or blocks its translation. In *MECP2*-deficient cells, where these regulatory miRNAs are expressed differently, the transgene is expressed more robustly.

The elegance of miRARE lies in its self-tuning behavior. In preclinical studies, AAV9/miniMECP2-miRARE restricted expression to only ~8% of neurons in wild-type mice, preventing overexpression toxicity (54). But in *Mecp2*-null mice, expression expanded to ~40% of neurons—sufficient to produce therapeutic benefit (54). Treated knockout mice showed delayed onset of motor abnormalities, improved gait, normalized breathing, and significantly prolonged survival. Importantly, the system appeared to adapt expression levels to cellular need, with cells already expressing adequate MeCP2 suppressing the transgene while deficient cells permitted robust expression. In non-human primates, lumbar intrathecal administration resulted in broad and uniform biodistribution throughout the brain and spinal cord, comparable to that achieved with intracisternal magna delivery (55).

##### 4.1.1.2 Clinical development: the REVEAL trials

miniMECP2 with miRARE technology is being further investigated, with the REVEAL clinical development program comprising two Phase 1/2/3 studies (NCT06152237 and NCT05606614). Both are open-label, multicenter trials with Part A dose-escalation and Part B dose-expansion components designed to evaluate the safety, tolerability, and preliminary efficacy of intrathecally administered TSHA-102 (56, 57).

##### 4.1.1.3 REVEAL pediatric study — eligibility and treatment

The REVEAL Pediatric Study enrolled females aged 5–8 years with genetically confirmed classical RTT due to pathogenic *MECP2* variants, a Clinical Global Impression–Severity (CGI-S) score ≥4, and no need for mechanical ventilation. Participants received a single intrathecal bolus via lumbar puncture at either 5.7 × 10^14^ or 1 × 10^15^ vector genomes. Immunosuppression, typically corticosteroids, begins 7 days before dosing to mitigate immune responses to the AAV vector. Post-treatment monitoring extends up to 6 years.

Primary endpoints focus on safety—incidence and severity of adverse events (AEs), serious AEs, and events of special interest including immune-mediated reactions, hepatotoxicity, neurologic worsening, and seizure exacerbation. Secondary endpoints evaluate preliminary efficacy using validated and exploratory measures including CGI-I/CGI-S scales, the Revised Motor Behavior Assessment (R-MBA), the Mullen Scales of Early Learning adapted for RTT (MSEL-A), Observer-Reported Communication Ability (ORCA), quantitative EEG metrics (auditory/visual evoked potentials), and monthly seizure frequency.

##### 4.1.1.4 Part A results: safety and preliminary efficacy

Topline Part A results released in 2024 demonstrated a favorable safety profile. TSHA-102 was well tolerated at both dose levels, with no treatment-related SAEs or dose-limiting toxicities (58). Most treatment-emergent AEs were mild to moderate and included transient elevations of liver transaminases (AST/ALT), pyrexia, and lethargy. Transaminase elevations were generally asymptomatic and responsive to steroid therapy. Temporary increases in cerebrospinal fluid neurofilament light chain (NfL) were observed in some participants but were not associated with neurologic decline. Notably, seizure control was maintained or improved following TSHA-102 administration. Enrollment of six participants in Part A is complete.

Preliminary efficacy exceeded natural-history expectations. All six participants achieved meaningful functional gains, with each acquiring or regaining at least one developmental milestone considered highly unlikely to occur spontaneously based on published natural-history data (<6.7% milestone acquisition in girls ≥6 years) (Table 6). Improvements spanned communication, fine-motor, and gross-motor domains—for example, responding to familiar names, following commands, using pincer grasp., self-feeding, pulling to stand, walking independently, and climbing stairs (59). Improvements emerged within 3–6 months and were sustained with continued follow-up. Standardized scale-based outcomes (MSEL-A, R-MBA, ORCA) corroborated these findings, and blinded centralized review of video evidence confirmed milestone achievements.

**Table 6:** Developmental skills/milestones by functional category.

Fine motor/Hand function	Gross motor	Communication/Social
Reached for toy	Sat with support when placed	Responded to familiar names or words
Taken a drink from a cup held without assistance	Sat without support when placed	Followed a command with a gesture
Used raking grasp to retrieve an object	Came to sitting	Followed a command without a gesture
Used a pincer grasp (either refined or modified)	Pulled to standing	Pointed for something they want
Finger fed	Stood while holding on	Waved bye-bye
Transferred an object from one hand to the other	Stood independently	Babbled
Used a spoon/fork to eat without assistance	Cruised around furniture or while holding someone’s hand	Used words with meaning
	Walked independently	Spoken in phrases (two words or more with meaning)
	Climbed up stairs with help	
	Climbed up stairs without help	
	Climbed down stairs with help	
	Climbed down stairs without help	
	Ran 10 feet without falling	

A clear dose–response relationship was observed: participants receiving 1 × 10^15^ vg demonstrated faster and more pronounced functional gains than those receiving 5.7 × 10^14^ vg. This supports biological plausibility and informed dose selection for Part B.

##### 4.1.1.5 Part B (pivotal expansion) — ongoing

The ongoing REVEAL pivotal expansion trial (NCT05606614) will enroll 15 females aged 6 to <22 years with typical RTT to further characterize safety and efficacy in a broader, more heterogeneous population. As of the May 2025 data cut, 12 participants (low dose n = 4; high dose n = 8) had received TSHA-102, representing a diverse range of *MECP2* variants and clinical severities (60).

Safety outcomes remain consistent with Part A (60). Efficacy data (*N* = 10) reflect cumulative functional improvement (60). Participants achieved a total of 22 developmental milestones, as verified through independent, blinded video adjudication using prespecified milestone definitions. In addition, 165 incremental skill gains were recorded across validated scales. At ≥9 months post-treatment, high-dose recipients consistently outperformed the low-dose cohort across multiple domains, demonstrating deepening dose-dependent benefit over time. Mean R-MBA score improved by −11.5 points in the low-dose cohort and −18.0 in the high-dose cohort. Mean CGI-I scores at ≥9 months were 2.8 and 1.0 for low- and high-dose cohorts, respectively. CGI-S improvement was recorded in 25% of low-dose and 33% of high-dose recipients at latest follow-up.

#### 4.1.2 NGN-401: full-length MECP2 with EXACT technology

##### 4.1.2.1 Molecular design and preclinical foundation

**Figure 2:**
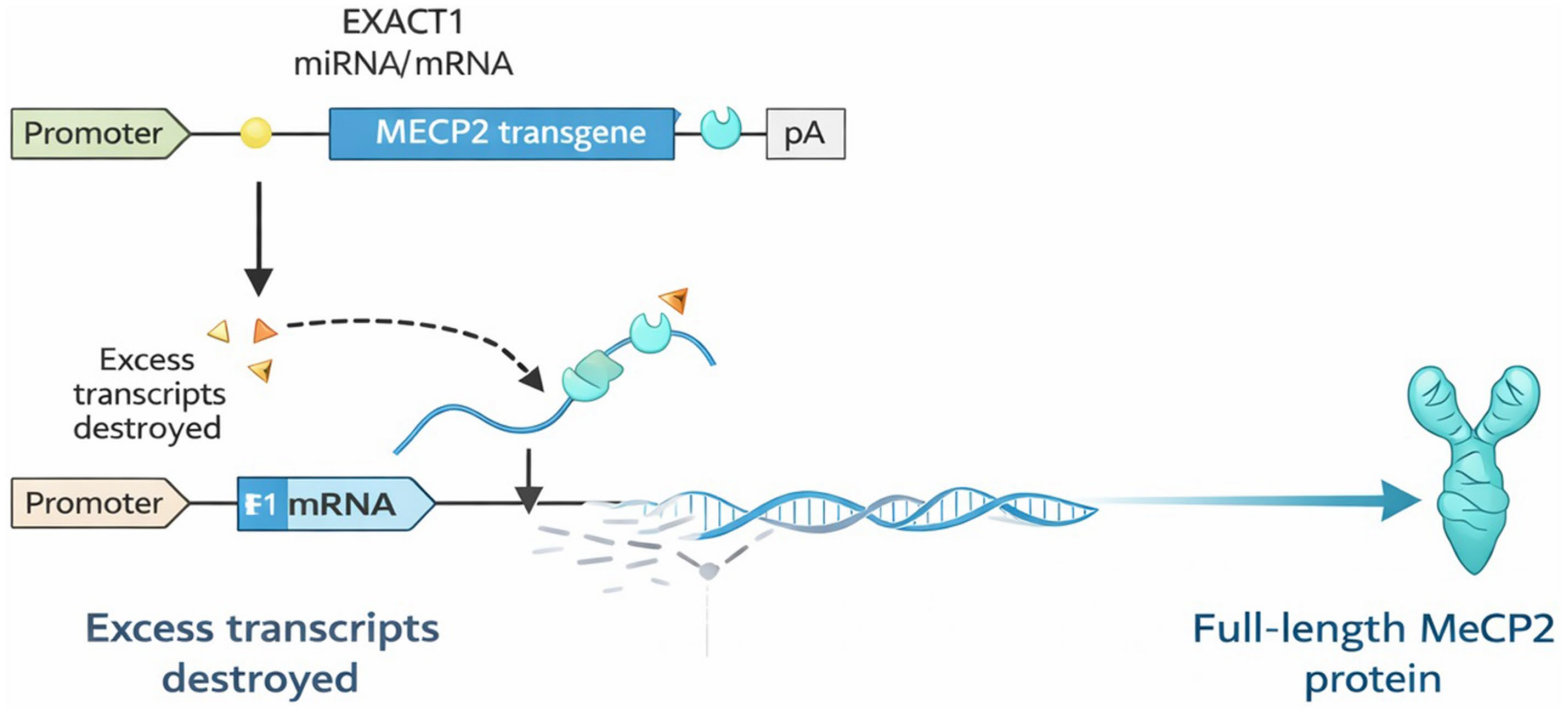
EXACT-mediated regulation of MECP2 transgene expression. The MECP2 transgene (blue) is transcribed into mRNA (blue lines) containing EXACT1 microRNA binding sites in the 3’UTR (pale blue symbols). A portion of the transcripts is translated into full-length MeCP2 protein (right). To prevent overexpression, EXACT1 microRNA (yellow/orange triangles) is co-expressed in proportion to the transgene. EXACT1 binds to its target sites on MECP2 transcripts, selectively degrading excess mRNA (left), thereby maintaining physiologic MeCP2 protein levels.

NGN-401 (Neurogene Inc.) takes a conceptually distinct approach: it delivers the full-length human *MECP2* gene rather than a truncated version, employing a novel self-regulating circuit called EXACT (Expression Attenuation via Construct Tuning) to control expression levels (Figure 2).

The EXACT system implements a synthetic, cell-autonomous, incoherent feedforward loop (61). A single ubiquitous promoter drives transcription of both the *MECP2* coding sequence and a designed, non-mammalian microRNA (EXACT1) (61). EXACT1 is engineered to recognize complementary target sites inserted into the 3′UTR of the same MECP2 transcript, creating an intrinsic negative feedback mechanism. When MECP2 expression rises, increased production of EXACT1 microRNA suppresses MECP2 translation; when expression falls, reduced EXACT1 levels relieve this repression. This design produces a dose-independent, self-buffering circuit that stabilizes cellular MECP2 abundance regardless of viral genome copy number.

Preclinical validation demonstrated the utility of this approach. In cell-based assays, EXACT-regulated constructs exhibited a significantly narrower distribution of MeCP2 protein levels across cells compared to conventional unregulated constructs (61). Importantly, the degree of regulation increased at higher transgene doses—precisely when the risk of overexpression toxicity is greatest. In *Mecp2*−/y hemizygous male mice, intracerebroventricular (ICV) administration of NGN-401 at postnatal day 1–2 resulted in prolonged survival (from ~10 weeks to >1 year) and amelioration of RTT-like motor, respiratory, and behavioral phenotypes (61). Critically, NGN-401 was well tolerated in female *Mecp2*+/− mice (the model more relevant to human RTT given mosaicism) and healthy juvenile non-human primates without causing the toxicity seen with unregulated *MECP2* overexpression constructs (61).

##### 4.1.2.2 Route of administration: ICV vs. intrathecal

A significant differentiator of NGN-401 is its intended route of administration: ICV injection rather than intrathecal lumbar puncture. Recent preclinical studies in nonhuman primates (juvenile cynomolgus macaques) compared ICV versus intrathecal lumbar (IT-L) delivery. ICV administration achieved markedly superior AAV biodistribution throughout the brain, with 10–100 × higher transgene RNA expression in cortical regions, deep brain structures, and upper spinal cord compared to equivalent or even higher IT-L doses (62). Importantly, systemic (liver) exposure was comparable between routes, indicating no liver-sparing advantage of IT-L delivery and suggesting similar systemic biodistribution despite the different injection sites (62). ICV delivery requires neurosurgical placement of a catheter into the lateral ventricle—a more invasive procedure than lumbar puncture but one routinely performed for ventriculoperitoneal shunt placement in pediatric neurosurgery centers. The improved CNS biodistribution may translate to enhanced efficacy, but it also necessitates greater neurosurgical expertise and infrastructure.

##### 4.1.2.3 Clinical development: the EMBOLDEN trial

NGN-401 is being evaluated in the EMBOLDEN (previously ASPIRE) trial (NCT05898620), initially designed as a Phase 1/2 dose-escalation study that has now transitioned to a pivotal, open-label, baseline-controlled, multicenter, single-arm efficacy trial.

Eligibility criteria include females with genetically confirmed typical RTT and a documented pathogenic *MECP2* mutation, stable antiseizure medication regimen for ≥12 weeks, post-regression stage, and either trofinetide-naïve status or prior discontinuation of trofinetide. The trial originally enrolled participants into sequential arms by age: Arms 1 and 2 (females aged 4–10 years) and Arm 3 (females ≥11 years) tested escalating doses. Arm 4, the pivotal cohort, enrolls females aged ≥3 years.

The primary efficacy endpoint is the proportion of responders defined by: (1) achieving a Clinical Global Impression-Improvement (CGI-I) score ≤3 (“minimally improved” or better) AND (2) gaining at least one developmental milestone from a predefined list of 28 skills across fine motor, gross motor, and communication/social domains, assessed via standardized video recordings independently reviewed by blinded central raters over 52 weeks. This composite endpoint ensures that both objective skill acquisition and global clinical meaningfulness are captured. A total of 33 participants will be evaluated for efficacy and safety over a three-year post-treatment period, with planned enrollment in a subsequent 12-year long-term follow-up study.

##### 4.1.2.4 Early clinical results: efficacy and safety signals

Early results from the low-dose cohort (1 × 10^15^ vg) demonstrated a favorable safety profile and encouraging efficacy (63). The first four treated girls achieved meaningful functional gains, acquiring 23 developmental skills collectively. Improvements spanned all functional categories: fine motor (reaching, grasping, finger feeding, utensil use), gross motor (sitting, standing, walking, climbing stairs), and communication (responding to names, following commands, pointing, waving, babbling, using words with meaning).

These gains, which would be exceedingly rare based on natural history (<6.7% spontaneous milestone acquisition rate), occurred early post-treatment and were sustained through ongoing follow-up. Clinician and caregiver assessments consistently rated participants as improved, supporting the clinical meaningfulness of observed changes.

##### 4.1.2.5 The high-dose safety event

A participant treated with the high dose (3 × 10^15^ vg) developed systemic hyperinflammatory syndrome several days post-dosing, characterized by fever, cytopenia, markedly elevated ferritin, and multi-organ dysfunction consistent with hemophagocytic lymphohistiocytosis (HLH) or macrophage activation syndrome (MAS) (64). Despite aggressive treatment with corticosteroids and immunosuppressive therapy, the participant succumbed to complications of the inflammatory syndrome. Two other participants had previously received the same 3 × 10^15^ vg dose without developing hyperinflammatory reactions, suggesting that individual susceptibility factors—such as subclinical infection, immune priming, or a genetic predisposition to HLH—may have contributed, rather than dose-dependent toxicity alone. However, high-dose treatments were immediately discontinued, and the maximum allowable dose was capped at 1 × 10^14^ vg/kg, with no participant receiving more than 1 × 10^15^ vg in total. Additional safety measures were implemented to reduce the risk of hyperinflammatory complications. Infection screening was strengthened to exclude any systemic illness within 30 days of dosing, including testing for EBV and CMV, and to rule out COVID-19 within 6 weeks. Before dosing, sites are now required to have anakinra (an IL-1 receptor antagonist and first-line therapy for HLH/MAS) immediately available and to coordinate with local HLH specialists. Post-dosing surveillance was intensified with daily assessments during the first week, including ferritin, fever, and blood counts to monitor for the classical HLH triad. A stepwise treatment algorithm was added, beginning with high-dose corticosteroids and escalating to anakinra for refractory cases. HLH-specific treatment pathways were also formally integrated into the protocol.

Both TSHA-102 and NGN-401 represent scientifically sophisticated attempts to overcome the unique challenges of RTT gene therapy, but they differ in key aspects (Table 7). Both programs have demonstrated proof-of-concept efficacy—functional gains across multiple domains that far exceed natural history expectations. The critical questions moving forward include: (1) whether full-length MECP2 (NGN-401) offers advantages over miniMECP2 (TSHA-102) in terms of functional protein activity; (2) whether ICV delivery’s superior biodistribution translates to greater clinical efficacy; (3) whether the enhanced safety protocols for NGN-401 adequately mitigate hyperinflammatory risk; and (4) what the optimal timing and patient selection criteria are for each approach.

**Table 7:** Comparative considerations: TSHA-102 vs. NGN-401.

Feature	TSHA-102	NGN-401
MECP2 construct	Truncated miniMECP2 (MBD + NID domains only)	Full-length human MECP2
Regulatory system	miRARE (endogenous miRNA-responsive)	EXACT (synthetic miRNA feedforward loop)
AAV type	Self-complementary AAV9	Standard single-stranded AAV9
Administration route	Intrathecal lumbar puncture	Intracerebroventricular (ICV)
Procedural complexity	Moderate (lumbar puncture)	Higher (neurosurgical ICV access)
CNS biodistribution	Moderate (based on IT delivery)	Superior (10–100 × higher in preclinical NHP studies)
Clinical stage	Phase 1/2 Part A complete; Part B pivotal enrolling	Phase 1/2 transitioning to pivotal; Arm 4 enrolling
Safety profile to date	Favorable; no SAEs or DLTs	Favorable at low doses; high-dose hyperinflammatory fatality
Efficacy signals	All participants gained ≥1 milestone; sustained improvements	Low-dose: 23 skills across 4 participants; sustained gains

MBD, methyl-CpG–binding domain; NID, NCoR/SMRT-interaction domain; miRARE, microRNA-responsive autoregulatory element; EXACT, expression-attenuating CRISPR-tuned circuit; AAV, adeno-associated virus; IT, intrathecal; ICV, intracerebroventricular; CNS, central nervous system; NHP, nonhuman primate; SAE, serious adverse event; DLT, dose-limiting toxicity.

## 5 Emerging and next-generation approaches

**Figure 3:**
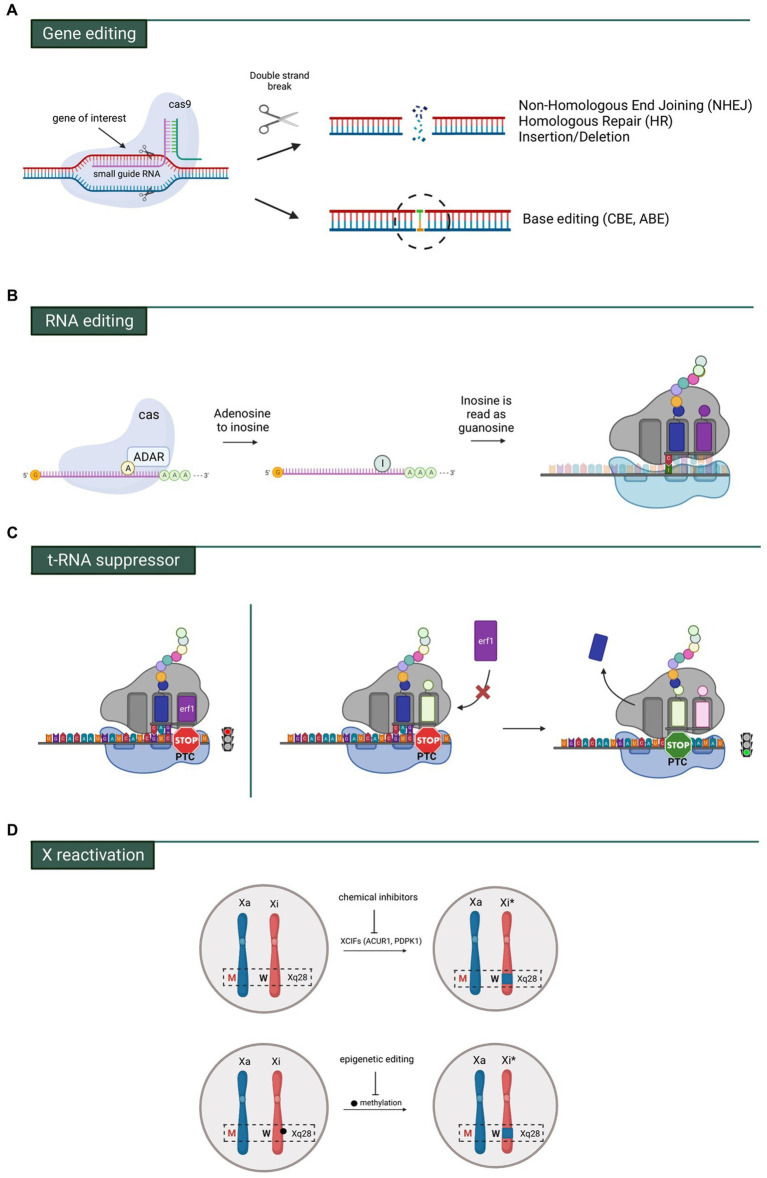
Innovative genetic approaches being explored for Rett syndrome: **(A)** gene editing using CRISPR-Cas9, which enables either broad modifications in DNA segments or precise single-nucleotide changes. **(B)** RNA editing via the ADAR system, which converts adenosine into inosine through deamination; inosine is interpreted as guanosine and pairs with cytosine in the tRNA anticodon. **(C)** Left panel: a premature termination codon (PTC) halts mRNA translation. Right panel: a specialized suppressor tRNA binds the PTC, permitting translation to continue. *Erf1* refers to eukaryotic translation termination factor 1. **(D)** The mutant *MECP2* allele (red M) resides on the active X chromosome (Xa), while the wild-type allele (black W) is on the inactive X chromosome (Xi). Reactivation of the wild-type allele (Xi*) can be achieved through chemical inhibition of silencing factors or epigenetic modulation, such as blocking DNA methylation. Reproduced from Palmieri et al. (95), under a Creative Commons Attribution License (CC BY 4.0).

Beyond AAV-mediated gene replacement, several innovative therapeutic strategies are progressing through preclinical development, each offering distinct advantages and facing unique challenges (Figure 3).

### 5.1 CRISPR-Cas9 genome editing

CRISPR-Cas9 genome editing offers the advantage of directly repairing pathogenic MECP2 mutations at the DNA level, providing the potential for permanent correction without the need for continuous transgene expression (65). The system uses a guide RNA to direct the Cas9 nuclease to a specific genomic site, where a double-strand break is introduced and subsequently repaired by non-homologous end joining or homology-directed repair using a supplied template (66).

Proof-of-concept work has shown successful correction of MECP2 mutations in patient-derived induced pluripotent stem cells (iPSCs) with repair of mutation in high efficiency (67, 68). Clinical translation is being pursued through the MECPer-3D program (NCT05740761), which focuses on CRISPR-Cas9 correction of the four most common MECP2 hotspot mutations—c.473C > T (p.T158M), c.502C > T (p.R168X), c.763C > T (p.R255X), and c.916C > T (p.R306C). The program includes development of mutation-specific guide RNAs and repair templates, evaluation of editing efficiency and specificity in patient-derived cellular models, assessment of off-target events through whole-genome sequencing, and preclinical testing in mouse and potentially large-animal models. Key endpoints include the proportion of alleles successfully edited and the characterization of any off-target genomic changes.

Several barriers remain for clinical application, including effective delivery of CRISPR components across the central nervous system, achieving sufficient editing efficiency to translate into meaningful clinical benefit, minimizing off-target effects, and ensuring safe dosage given the narrow therapeutic window of MeCP2 and the irreversible nature of genomic editing. Mosaic correction is also expected, as only transduced cells will be edited; however, even a shift toward a greater proportion of functional neurons may be beneficial. Despite these challenges, genome editing represents a promising long-term strategy because of its potential as a one-time, durable corrective treatment.

### 5.2 RNA editing with ADAR

An alternative to permanent DNA editing is transient correction at the RNA level using adenosine deaminases acting on RNA (ADARs). ADARs catalyze adenosine-to-inosine (A → I) editing in double-stranded RNA, and because inosine is interpreted as guanosine (G) during translation, this strategy enables correction of specific point mutations—including pathogenic G > A variants—directly at the mRNA level. Sinnamon et al. provided proof-of-concept for MECP2 RNA editing in mouse neurons by engineering an ADAR2 catalytic domain fused to an RNA-binding protein that guides editing to Mecp2 mRNA (69). This approach produced up to 72% editing efficiency, restored MeCP2 protein levels and heterochromatin localization, and improved electrophysiological properties and gene-expression patterns in edited neurons (69, 70).

RNA editing offers several advantages: its effects are reversible and tunable, requiring repeated dosing similar to ASOs; it poses a lower risk of off-target consequences compared with permanent DNA modification; and in principle it can treat multiple mutation types by redirecting ADAR activity to different transcript sites. However, key challenges remain, including achieving sufficient editing efficiency across enough neurons, optimizing CNS delivery for either repeated dosing or long-term AAV-mediated ADAR expression, controlling immunogenicity of engineered ADAR proteins, ensuring high target specificity, and addressing durability, as ongoing administration or sustained transgene expression is required. Although still in early preclinical development, RNA editing represents a promising middle ground between symptomatic treatment and irreversible genome editing, offering the potential to correct pathogenic MECP2 mutations without altering DNA permanently.

### 5.3 Translation readthrough for nonsense mutations

Approximately 30–35% of pathogenic *MECP2* variants are nonsense mutations that prematurely truncate the protein (71). Translation readthrough strategies aim to suppress these premature termination codons (PTCs), allowing ribosomes to produce near–full-length MeCP2. Two approaches are under investigation: small-molecule readthrough compounds, including aminoglycosides (e.g., gentamicin, G418) and designer drugs like PTC124 (ataluren), which alter ribosomal fidelity to incorporate an amino acid at the stop codon; and suppressor tRNAs engineered to recognize specific stop codons and insert an amino acid to enable continued translation (71–73).

While these strategies have shown promise in other genetic disorders such as Duchenne muscular dystrophy and cystic fibrosis, their adaptation to RTT faces significant challenges. Readthrough must be efficient enough to restore functional MeCP2 but selective enough to avoid suppressing normal stop codons across the genome, which could create off-target protein extensions. Moreover, the amino acid incorporated at the PTC may differ from the wild-type residue, potentially impacting protein function. MeCP2’s narrow dosage sensitivity further complicates translation readthrough, as even successful suppression must achieve expression within the therapeutic window. Translation readthrough for RTT remains in early discovery, with proof-of-concept studies ongoing in cellular models, and no clinical programs have yet been initiated.

### 5.4 X-chromosome reactivation: unlocking the endogenous wild-type allele

Perhaps the most conceptually elegant approach for female RTT patients is reactivation of the silenced wild-type *MECP2* allele on the inactive X chromosome (Xi) (74–76). Because females undergo random X-inactivation early in development, approximately half of neurons express the mutant allele while the remaining half carry a functional, but epigenetically silenced, MECP2 copy. Selectively reactivating this Xi-linked wild-type allele—without globally disrupting X-chromosome dosage compensation—could restore physiological MeCP2 levels using the patient’s own endogenous gene.

Earlier work also demonstrated that combined inhibition of ACVR1 and PDPK1, two regulators of X-chromosome silencing, can reactivate Xi-linked Mecp2 in neurons and achieve partial *in vivo* reactivation in heterozygous female mouse brains (77, 78). Large-scale functional screens have identified multiple regulatory pathways that control MECP2 silencing on the inactive X chromosome (Xi). An RNAi screen in RTT patient–derived cells revealed that components of the BMP/TGF-*β* signaling pathway modulate expression of XIST, the long noncoding RNA that governs X-inactivation; inhibiting this pathway partially reactivated Xi-linked MECP2 without inducing global X-chromosome reactivation (79). Follow-up studies showed that small-molecule BMP inhibitors can similarly restore MECP2 expression in primary neurons from Mecp2-heterozygous female mice, providing pharmacologic proof-of-concept (78).

Another study demonstrated that pairing an antisense oligonucleotide (ASO) targeting Xist RNA with the DNA methylation inhibitor 5-azacytidine (5-Aza) produced robust reactivation of a silent Mecp2 luciferase reporter in mouse fibroblasts, highlighting the need to simultaneously disrupt RNA-based and DNA-based silencing layers (75). High-throughput screening also identified JAK/STAT pathway inhibitors (AG490, Jaki) as additional modulators of Xi stability, although reactivation effects were strongly cell-type dependent—effective in mouse fibroblasts but limited in human Xi-containing cell lines (80).

More recently, a genome-wide CRISPR loss-of-function screen in female fibroblasts identified miR-106a as a key regulator of the RNA-based silencing machinery that stabilizes Xi (81). Inhibiting miR-106a disrupted this system, destabilized the inactive X chromosome, and produced therapeutic benefits in RTT mouse models—extending survival, improving motor and exploratory behaviors, and reducing breathing abnormalities (81).

To achieve gene-specific reactivation without globally perturbing X-chromosome dosage, targeted epigenetic editing has emerged as a promising strategy. One study used dCas9–Tet1–mediated promoter demethylation to selectively reactivate the Xi-linked MECP2 allele in human embryonic stem cell–derived neurons, achieving up to 82% restoration of MeCP2 protein with minimal off-target effects (82). Similar approaches reactivated Xi-linked MECP2-GFP in neurons at more modest levels (~18%) but confirmed allele specificity without widespread reactivation of other X-linked genes (82). However, the large size of dCas9–Tet1 poses significant challenges for AAV-mediated CNS delivery *in vivo*.

Together, these studies demonstrate that X-chromosome reactivation represents a compelling therapeutic avenue capable of restoring endogenous MECP2 expression, avoiding the immunogenicity and dosage risks of gene-addition therapies, and potentially enabling small-molecule or ASO-based clinical approaches. Key challenges remain—including achieving brain-wide and allele-specific reactivation, ensuring CNS penetrance of candidate therapies, avoiding unintended activation of other X-linked genes, and determining whether continuous treatment will be required. Nonetheless, ongoing dissection of the druggable regulatory nodes governing Xi maintenance continues to advance this strategy toward translational feasibility.

## 6 Critical considerations for clinical translation

### 6.1 Optimal therapeutic window and timing of intervention

A central question for clinical translation is when gene therapy should be delivered. Mouse studies show that restoring MeCP2 can be effective across a wide age range—from presymptomatic neonates to fully symptomatic adults—indicating that the Rett phenotype remains at least partly reversible even after prolonged deficiency. Nonetheless, several considerations favor earlier intervention: the first 2–3 years of life represent critical windows of synaptogenesis and circuit formation; prolonged MeCP2 deficiency may lead to structural neuronal and glial changes that become harder to reverse; and treating before or during early regression could prevent milestone loss rather than requiring later recovery (83, 84). Conversely, treatment of older individuals has advantages including greater diagnostic certainty, a clearer risk–benefit rationale in severely affected patients, and more straightforward outcome assessment. Current clinical trials enroll patients ages 3–20+, reflecting uncertainty about the optimal window. As safety becomes better defined, intervention in younger children—including presymptomatic infants identified by genetic screening—will likely be explored. Ultimately, a stratified approach may emerge in which early treatment maximizes developmental potential while older individuals are treated to achieve meaningful functional gains. In summary, while pharmacological agents like Trofinetide are effective across a broad age range (2 years to adult), emerging gene therapies such as TSHA-102 and NGN-401 (currently recruiting down to age 3 in pivotal trials) suggest a potential for functional ‘regain’ that is highly sensitive to the developmental window of administration.

### 6.2 Dosing, biodistribution, and the “enough but not too much” problem

Gene therapy for RTT requires dosing that is both effective and safe, balancing transduction thresholds with the risk of overexpression toxicity. It remains unclear what percentage of brain cells must express the transgene in humans, whether certain regions (e.g., cortex, brainstem) require preferential targeting, and how neuronal versus glial transduction contributes to benefit (83, 85). The fatal hyperinflammatory reaction observed with high-dose NGN-401 highlights that toxicity may arise not only from excessive MeCP2 but also from high systemic AAV loads. Administration route strongly influences biodistribution: ICV delivery (NGN-401) provides broad brain penetration but requires neurosurgery, whereas intrathecal lumbar injection (TSHA-102) is less invasive but may yield more limited forebrain distribution. Future approaches—such as optimized injection routes, convection-enhanced delivery, nanoparticle-based gene delivery systems, or next-generation AAV capsids with improved CNS tropism—may help navigate this therapeutic window (86, 87).

### 6.3 Measuring meaningful change

Efficacy assessment in RTT trials is challenging due to floor effects on standard developmental tests, lack of validated disease-specific tools, and the risk of bias in caregiver-reported outcomes. Recently developed milestone checklists address disease specificity but still require ongoing validation and regulatory acceptance. Video-based blinded raters improve objectivity but increase logistical complexity. Determining what constitutes clinically meaningful benefit is equally difficult; families report that even modest gains in independence, communication, or daily living skills are profoundly valuable. Natural history variability further complicates interpretation of single-arm studies, necessitating robust genotype-informed natural history datasets. Current trials use composite endpoints that combine milestone achievement with CGI-I scores, while multiple candidate biomarkers (EEG signatures, neuroimaging connectivity, eye-tracking, and brain-derived plasma microRNAs) may ultimately provide objective surrogate endpoints if validated against functional outcomes (88–94).

### 6.4 Long-term safety

Because gene therapy aims to deliver lifelong MeCP2 expression, long-term safety remains largely unknown. Open questions include durability of expression, feasibility of re-dosing in the context of anti-AAV immunity, potential for delayed toxicity from MeCP2 overexpression, risk of chronic CNS immune responses, and the theoretical possibility of insertional mutagenesis despite low AAV integration rates. Another consideration is whether restoring MeCP2 in a brain that developed under deficiency could introduce new vulnerabilities. Addressing these uncertainties will require decades-long surveillance; current protocols mandate 15-year follow-up for all treated individuals.

### 6.5 Access, equity, and economic barriers

Gene therapy for RTT is expected to cost in the range of other approved AAV therapies ($1–3 M+), raising questions about payer coverage even if treatment reduces long-term care costs. Access is further constrained by the need for specialized centers capable of administering therapy and monitoring for complications, disproportionately affecting families in rural areas, underserved communities, and low-income countries. Regulatory and reimbursement heterogeneity across regions will further influence global availability. Patient advocacy organizations have played a central role in advancing research, but ensuring equitable access after approval will require policy coordination, financing innovation, and commitment from manufacturers and healthcare systems.

### 6.6 Ethical considerations

Gene therapy trials in severely affected children raise complex ethical questions. Families must make decisions amid profound emotional pressure, uncertain benefit, and real risks—including catastrophic adverse events. Clinicians and researchers must communicate risks and uncertainties honestly, avoiding both pessimism that discourages participation and optimism that overstates benefit. Early-phase studies blur the boundary between research and therapy, especially when early improvement fuels calls for expanded access. Equity concerns also arise if transformative treatments ultimately reach only children with geographic or financial privilege.

## 7 Conclusion

RTT highlights both the promise and the complexity of precision medicine for neurodevelopmental disorders. In just over two decades, the field has moved from identifying *MECP2* as the causative gene to demonstrating phenotypic reversibility in animal models and now to evaluating multiple gene therapies in human trials. Trofinetide marked the first disease-specific therapy, while gene-replacement approaches such as TSHA-102 and NGN-401 hold the potential for far greater clinical impact and have already shown early signals of meaningful functional improvement in treated children. At the same time, enthusiasm must be balanced with caution. Serious adverse events, uncertainties surrounding optimal dosing, timing, durability, and long-term safety, and persistent barriers to access and affordability underscore the need for rigorous science and responsible translation. As these therapies advance, neurologists will play a central role in guiding families, supporting clinical trials, preparing systems for delivery, and advocating for equitable access. The coming years will determine whether decades of discovery culminate in transformative, widely accessible treatment for individuals with RTT. Achieving that goal will require continued innovation, sustained vigilance, and a commitment to ensuring that every affected individual has the opportunity to benefit from emerging therapeutic breakthroughs.

## References

[1] Nguengang Wakap S Lambert DM Olry A Rodwell C Gueydan C Lanneau V . Estimating cumulative point prevalence of rare diseases: analysis of the Orphanet database. Eur J Hum Genet. (2020) 28:165–73. doi: 10.1038/s41431-019-0508-0 31527858 PMC6974615

[2] Cardinalhealth. 2025. Advanced therapies report. Available online at: https://www.cardinalhealth.com/En/Services/Manufacturer/Biopharmaceutical/Cell-and-Gene-Therapies/Advanced-Therapies-Report.Html. [Accessed October 12, 2025].

[3] Jaitovich Groisman I Hurlimann T Shoham A Godard B. Practices and views of neurologists regarding the use of whole-genome sequencing in clinical settings: a web-based survey. Eur J Hum Genet. (2017) 25:801–8. doi: 10.1038/ejhg.2017.64, 28488681 PMC5520076

[4] Salm M Abbate K Appelbaum P Ottman R Chung W Marder K . Use of genetic tests among neurologists and psychiatrists: knowledge, attitudes, behaviors, and needs for training. J Genet Couns. (2014) 23:156–63. doi: 10.1007/s10897-013-9624-0, 23793969 PMC3812264

[5] Petriti U Dudman DC Scosyrev E Lopez-Leon S. Global prevalence of Rett syndrome: systematic review and Meta-analysis. Syst Rev. (2023) 12:5. doi: 10.1186/s13643-023-02169-6, 36642718 PMC9841621

[6] Percy AK Lane JB. Rett syndrome: clinical and molecular update. Curr Opin Pediatr. (2004) 16:670–7. doi: 10.1097/01.mop.0000143693.59408.ce, 15548931

[7] Rett A. On a unusual brain atrophy syndrome in Hyperammonemia in childhood. Wien Med Wochenschr. (1966) 116:723–6. 5300597

[8] Hagberg B Aicardi J Dias K Ramos O. A progressive syndrome of autism, dementia, Ataxia, and loss of purposeful hand use in girls: Rett's syndrome: report of 35 cases. Ann Neurol. (1983) 14:471–9. doi: 10.1002/ana.410140412, 6638958

[9] Amir RE den Van Veyver IB Wan M Tran CQ Francke U Zoghbi HY. Rett syndrome is caused by mutations in X-linked Mecp2, encoding methyl-Cpg-binding protein 2. Nat Genet. (1999) 23:185–8. doi: 10.1038/13810, 10508514

[10] Amir RE Zoghbi HY. Rett syndrome: methyl-Cpg-binding protein 2 mutations and phenotype-genotype correlations. Am J Med Genet. (2000) 97:147–52. doi: 10.1002/1096-8628(200022)97:2<147::aid-ajmg6>3.0.co;2-o, 11180222

[11] Hagberg B Goutières F Hanefeld F Rett A Wilson J. Rett syndrome: criteria for inclusion and exclusion. Brain Dev. (1985) 7:372–3. doi: 10.1016/s0387-7604(85)80048-6, 4061772

[12] Spagnoli C Fusco C Pisani F. Rett syndrome spectrum in monogenic developmental-epileptic encephalopathies and epilepsies: a review. Genes. (2021) 12. doi: 10.3390/genes12081157, 34440332 PMC8394997

[13] Vignoli A La Briola F Peron A Turner K Savini M Cogliati F . Medical care of adolescents and women with Rett syndrome: an Italian study. Am J Med Genet A. (2012) 158A:13–8. doi: 10.1002/ajmg.a.34367, 22139899

[14] Anderson A Wong K Jacoby P Downs J Leonard H. Twenty years of surveillance in Rett syndrome: what does this tell us? Orphanet J Rare Dis. (2014) 9:87. doi: 10.1186/1750-1172-9-87, 24942262 PMC4078387

[15] Pintaudi M Baglietto MG Gaggero R Parodi E Pessagno A Marchi M . Clinical and electroencephalographic features in patients with Cdkl5 mutations: two new Italian cases and review of the literature. Epilepsy Behav. (2008) 12:326–31. doi: 10.1016/j.yebeh.2007.10.010, 18063413

[16] Glaze DG Percy AK Skinner S Motil KJ Neul JL Barrish JO . Epilepsy and the natural history of Rett syndrome. Neurology. (2010) 74:909–12. doi: 10.1212/WNL.0b013e3181d6b852, 20231667 PMC2836870

[17] Corchón S Carrillo-López I Cauli O. Quality of life related to clinical features in patients with Rett syndrome and their parents: a systematic review. Metab Brain Dis. (2018) 33:1801–10. doi: 10.1007/s11011-018-0316-1 30220073

[18] Amin S Ruban-Fell B Newell I Evans J Vyas K Nortvedt C . Treatment guidelines for rare, early-onset conditions associated with epileptic seizures: a literature review on Rett syndrome and tuberous sclerosis complex. Orphanet J Rare Dis. (2024) 19:89. doi: 10.1186/s13023-023-02994-x, 38409029 PMC10895812

[19] Amoako AN Hare DJ. Non-medical interventions for individuals with Rett syndrome: a systematic review. J Appl Res Intellect Disabil. (2020) 33:808–27. doi: 10.1111/jar.12694, 31833197

[20] Percy AK Lane JB Childers J Skinner S Annese F Barrish J . Rett syndrome: north American database. J Child Neurol. (2007) 22:1338–41. doi: 10.1177/0883073807308715, 18174548

[21] Shen J Liu L Yang Y Zhou M Xu S Zhang W . Insulin-like growth factor 1 has the potential to be used as a diagnostic tool and treatment target for autism spectrum disorders. Cureus. (2024) 16:e65393. doi: 10.7759/cureus.65393, 39188438 PMC11346671

[22] Parent H Ferranti A Niswender C. Trofinetide: a pioneering treatment for Rett syndrome. Trends Pharmacol Sci. (2023) 44:740–1. doi: 10.1016/j.tips.2023.06.008, 37460385 PMC10529922

[23] Camillo L Pozzi M Bernardo P Pisano S Nobile M. Profile of trofinetide in the treatment of Rett syndrome: design, development and potential place in therapy. Drug Des Devel Ther. (2024) 18:5023–40. doi: 10.2147/dddt.S383133, 39525048 PMC11550706

[24] Neul JL Percy AK Benke TA Berry-Kravis EM Glaze DG Peters SU . Design and outcome measures of Lavender, a phase 3 study of trofinetide for Rett syndrome. Contemp Clin Trials. (2022) 114:106704. doi: 10.1016/j.cct.2022.106704, 35149233

[25] Neul JL Percy AK Benke TA Berry-Kravis EM Glaze DG Marsh ED . Trofinetide for the treatment of Rett syndrome: a randomized phase 3 study. Nat Med. (2023) 29:1468–75. Epub 20230608. doi: 10.1038/s41591-023-02398-1, 37291210 PMC10287558

[26] Neul JL Percy AK Benke TA Berry-Kravis EM Glaze DG Peters SU . Trofinetide treatment demonstrates a benefit over placebo for the ability to communicate in Rett syndrome. Pediatr Neurol. (2024) 152:63–72. doi: 10.1016/j.pediatrneurol.2023.11.005, 38232652

[27] Abbas A Fayoud AM El Din Moawad MH Hamad AA Hamouda H Fouad EA. Safety and efficacy of trofinetide in Rett syndrome: a systematic review and meta-analysis of randomized controlled trials. BMC Pediatr. (2024) 24:206. doi: 10.1186/s12887-024-04526-3, 38521908 PMC10960414

[28] Abo Zeid M Elrosasy A Mohamed RG Ghazou A Goufa E Hassan N . A meta-analysis of the efficacy and safety of trofinetide in patients with Rett syndrome. Neurol Sci. (2024) 45:4767–78. doi: 10.1007/s10072-024-07584-8, 38771525 PMC11422260

[29] Percy AK Ryther R Marsh ED Neul JL Benke TA Berry-Kravis EM . Results from the phase 2/3 Daffodil study of Trofinetide in girls aged 2-4 years with Rett syndrome. Med. (2025) 6:608. doi: 10.1016/j.medj.2025.100608, 40043705

[30] Percy AK Neul JL Benke TA Berry-Kravis EM Glaze DG Marsh ED . Trofinetide for the treatment of Rett syndrome: long-term safety and efficacy results of the 32-month, open-label Lilac-2 study. Med. (2024) 5:1275–81.e2. doi: 10.1016/j.medj.2024.06.007, 39025065

[31] Percy AK Neul JL Benke TA Berry-Kravis EM Glaze DG Marsh ED . Trofinetide for the treatment of Rett syndrome: results from the open-label extension Lilac study. Med. (2024) 5:1178–89.e3. doi: 10.1016/j.medj.2024.05.018, 38917793

[32] Cosand L Mayman H Downs J Abler V. Real-world benefits and tolerability of trofinetide for the treatment of Rett syndrome: the Lotus study. Dev Med Child Neurol. (2025) 11:16482. doi: 10.1111/dmcn.16482 PMC12875184 40936177

[33] Guy J Gan J Selfridge J Cobb S Bird A. Reversal of neurological defects in a mouse model of Rett syndrome. Science. (2007) 315:1143–7. doi: 10.1126/science.1138389, 17289941 PMC7610836

[34] Bassuk AG. Gene therapy for Rett syndrome. Genes Brain Behav. (2022) 21:e12754. doi: 10.1111/gbb.12754, 34053173 PMC9744469

[35] Singh J Goodman-Vincent E Santosh P. Evidence synthesis of gene therapy and gene editing from different disorders-implications for individuals with Rett syndrome: a systematic review. Int J Mol Sci. (2023) 24:109023. doi: 10.3390/ijms24109023, 37240368 PMC10219055

[36] Brand BA Blesson AE Smith-Hicks CL. The impact of X-chromosome inactivation on phenotypic expression of X-linked neurodevelopmental disorders. Brain Sci. (2021) 11:1070904. doi: 10.3390/brainsci11070904, 34356138 PMC8305405

[37] Braunschweig D Simcox T Samaco RC LaSalle JM. X-chromosome inactivation ratios affect wild-type Mecp2 expression within mosaic Rett syndrome and Mecp2−/+ mouse brain. Hum Mol Genet. (2004) 13:1275–86. doi: 10.1093/hmg/ddh142, 15115765

[38] Cheung AY Horvath LM Carrel L Ellis J. X-chromosome inactivation in Rett syndrome human induced pluripotent stem cells. Front Psych. (2012) 3:24. doi: 10.3389/fpsyt.2012.00024, 22470355 PMC3311266

[39] Takagi N. The role of X-chromosome inactivation in the manifestation of Rett syndrome. Brain Dev. (2001) 23 Suppl 1:S182–5. doi: 10.1016/s0387-7604(01)00362-x, 11738869

[40] Young JI Zoghbi HY. X-chromosome inactivation patterns are unbalanced and affect the phenotypic outcome in a mouse model of Rett syndrome. Am J Hum Genet. (2004) 74:511–20. doi: 10.1086/382228, 14973779 PMC1182264

[41] Ak M Suter B Akturk Z Harris H Bowyer K Mignon L . Exploring the characteristics and most bothersome symptoms in Mecp2 duplication syndrome to pave the path toward developing parent-oriented outcome measures. Mol Genet Genomic Med. (2022) 10:e1989. doi: 10.1002/mgg3.1989, 35702943 PMC9356562

[42] Webb T Watkiss E. A comparative study of X-inactivation in Rett syndrome Probands and control subjects. Clin Genet. (1996) 49:189–95. doi: 10.1111/j.1399-0004.1996.tb03285.x, 8828984

[43] Rastegar M Hotta A Pasceri P Makarem M Cheung AY Elliott S . Mecp2 isoform-specific vectors with regulated expression for Rett syndrome gene therapy. PLoS One. (2009) 4:e6810. doi: 10.1371/journal.pone.0006810, 19710912 PMC2728539

[44] Gadalla KK Bailey ME Spike RC Ross PD Woodard KT Kalburgi SN . Improved survival and reduced phenotypic severity following Aav9/Mecp2 gene transfer to neonatal and juvenile male Mecp2 knockout mice. Mol Ther. (2013) 21:18–30. doi: 10.1038/mt.2012.200, 23011033 PMC3536818

[45] Gadalla KKE Vudhironarit T Hector RD Sinnett S Bahey NG Bailey MES . Development of a novel Aav gene therapy cassette with improved safety features and efficacy in a mouse model of Rett syndrome. Mol Ther Methods Clin Dev. (2017) 5:180–90. doi: 10.1016/j.omtm.2017.04.007, 28497075 PMC5423329

[46] Garg SK Lioy DT Cheval H McGann JC Bissonnette JM Murtha MJ . Systemic delivery of Mecp2 rescues behavioral and cellular deficits in female mouse models of Rett syndrome. J Neurosci. (2013) 33:13612–20. doi: 10.1523/jneurosci.1854-13.2013, 23966684 PMC3755711

[47] Matagne V Ehinger Y Saidi L Borges-Correia A Barkats M Bartoli M . A codon-optimized Mecp2 transgene corrects breathing deficits and improves survival in a mouse model of Rett syndrome. Neurobiol Dis. (2017) 99:1–11. doi: 10.1016/j.nbd.2016.12.009, 27974239

[48] Matagne V Borloz E Ehinger Y Saidi L Villard L Roux JC. Severe offtarget effects following intravenous delivery of Aav9-Mecp2 in a female mouse model of Rett syndrome. Neurobiol Dis. (2021) 149:105235. doi: 10.1016/j.nbd.2020.105235, 33383186

[49] Yang K Cheng C Yuan Y Zhang Y Shan S Qiu Z. Extension of the lifespan of a mouse model of Rett syndrome by intracerebroventricular delivery of Mecp2. Neurosci Bull. (2023) 39:297–302. doi: 10.1007/s12264-022-00974-y, 36374470 PMC9905459

[50] Luoni M Giannelli S Indrigo MT Niro A Massimino L Iannielli A . Whole brain delivery of an instability-prone Mecp2 transgene improves behavioral and molecular pathological defects in mouse models of Rett syndrome. eLife. (2020) 9. doi: 10.7554/eLife.52629, 32207685 PMC7117907

[51] Garg N Zhou Z Marsh ED Niswender CM Pichard DC. Irsf 2023 - Rett syndrome scientific meeting report. Transl Sci Rare Dis. (2023) 6:137–50. doi: 10.3233/trd-230063

[52] Tillotson R Selfridge J Koerner MV Gadalla KKE Guy J De Sousa D . Radically truncated Mecp2 rescues Rett syndrome-like neurological defects. Nature. (2017) 550:398–401. doi: 10.1038/nature24058, 29019980 PMC5884422

[53] Sadhu C Lyons C Oh J Jagadeeswaran I Gray SJ Sinnett SE. The efficacy of a human-ready mini MECP2 gene therapy in a pre-clinical model of Rett syndrome. Genes (2023) 15:31. doi: 10.3390/genes15010031 PMC10815157 38254921

[54] Sinnett SE Boyle E Lyons C Gray SJ. Engineered Microrna-based regulatory element permits safe high-dose Minimecp2 gene therapy in Rett mice. Brain. (2021) 144:3005–19. doi: 10.1093/brain/awab182, 33950254 PMC8783608

[55] Emdadul Haque SN Nino D Rumana H-A Sean M Alain L Fred P, Raav9 vector biodistribution in brain and spinal cord via lumbar intrathecal infusion in nonhuman Primates (Nhp): assessing the administration route leveraged in Tsha-102 Rett syndrome clinical trials. International Rett Syndrome Foundation (IRSF) Rett Syndrome Scientific Meeting; 2025. Texas: IRSF

[56] Meglio M. Promising phase 1/2 data released on Rett syndrome gene therapy agent Tsha-102. NeurologyLive (2023). Available online at: https://www.neurologylive.com/view/promising-phase-1-2-data-released-rett-syndrome-gene-therapy-agent-tsha-102 (Accessed December 10, 2025).

[57] Stansfield N Ciccone I. Second patient dosing recommended for Tsha-102 gene therapy trial in Rett syndrome. NeurologyLive. (2023). Available online at: https://www.neurologylive.com/view/second-patient-dosing-recommended-tsha-102-gene-therapy-trial-rett-syndrome (Accessed December 10, 2025).

[58] Nagendran S. (2025). First-cohort data from the Reveal adolescent/adult and pediatric studies of Tsha-102 gene therapy for Rett syndrome. 9th World Rett Syndrome Congress. Texas: IRSF

[59] Keeley Jessica Berry-Kravis Elizabeth Lieberman David Phillips Kristin Karbocus Chelsea Pisani Laura . Every gain, expanding possibilities: caregiver insights on meaningful improvement in Rett syndrome gene therapy. International Rett Syndrome Foundation (IRSF) Rett Syndrome Scientific Meeting; 2025. Texas: IRSF

[60] Elsa Rossignol EB-K Haas Richard Ryther Robin Downs Jenny Meijer Inge Clarkson Tessa . Achieving developmental milestones and broad restoration of function in Rett syndrome: the potential of Tsha-102 gene therapy. 54th Child Neurology Society Annual Meeting; 2025. Charlotte: Childneurologysociety

[61] Ross PD Gadalla KKE Thomson SR Selfridge J Bahey NG Benito J . Self-regulating gene therapy ameliorates phenotypes and overcomes gene dosage sensitivity in a mouse model of Rett syndrome. Sci Transl Med. (2025) 17:eadq3614. doi: 10.1126/scitranslmed.adq3614, 40173263

[62] Burstein Suzanne R. Gonzalez Solange Cram Rebecca Biezonski Dominik Proetzel Gabriele Albanis Effie . Intracerebroventricular (Icv) delivery of Ngn-401 drives superior transgene expression to Key areas of the brain when compared to lumbar intrathecal (it-L) delivery at a clinically relevant dose – implications for a one-time gene therapy for Rett syndrome. European Society of Gene & Cell Therapy (ESGCT) Annual Congress, 2025. New York: Neurogene Inc.

[63] Suter Bernhard Benke Timothy Ellaway Carolyn Neul Jeffrey Jaggumantri Sravan Feng Cynthia . Ngn-401, a novel regulated gene therapy for Rett syndrome: preliminary results from the first-in-human study. 2024 Child Neurology Society 53rd Annual Meeting; 2024. New York: Neurogene Inc.

[64] Donald Aimee Byrne Barry J. Cron Randy Q. Albanis Effie Cobb Stuart Jordan Julie, Hemophagocytic Lymphohistiocytosis (Hlh)/Hyperinflammatory syndrome following high dose Aav9 therapy. 2025 IRSF Rett Syndrome Scientific Meeting; 2025. Texas: IRSF

[65] Jiang F Doudna JA. Crispr–Cas9 structures and mechanisms. Annu Rev Biophys. (2017) 46:505–29. doi: 10.1146/annurev-biophys-062215-010822, 28375731

[66] Ran FA Hsu PD Lin C-Y Gootenberg JS Konermann S Trevino AE . Double nicking by Rna-guided Crispr Cas9 for enhanced genome editing specificity. Cell. (2013) 154:1380–9. doi: 10.1016/j.cell.2013.08.021, 23992846 PMC3856256

[67] Le TTH Tran NT Dao TML Nguyen DD Do HD Ha TL . Efficient and precise Crispr/Cas9-mediated Mecp2 modifications in human-induced pluripotent stem cells. Front Genet. (2019) 10:625. doi: 10.3389/fgene.2019.00625, 31333716 PMC6614930

[68] Coorey B Haase F Ellaway C Clarke A Lisowski L Gold WA. Gene editing and Rett syndrome: does it make the cut? CRISPR J. (2022) 5:490–9. doi: 10.1089/crispr.2022.0020, 35881862

[69] Sinnamon JR Kim SY Corson GM Song Z Nakai H Adelman JP . Site-directed Rna repair of endogenous Mecp2 Rna in neurons. Proc Natl Acad Sci USA. (2017) 114:E9395–e402. doi: 10.1073/pnas.1715320114, 29078406 PMC5676935

[70] Sinnamon JR Jacobson ME Yung JF Fisk JR Jeng S McWeeney SK . Targeted Rna editing in brainstem alleviates respiratory dysfunction in a mouse model of Rett syndrome. Proc Natl Acad Sci USA. (2022) 119:e2206053119. doi: 10.1073/pnas.2206053119, 35939700 PMC9388114

[71] Lebeda D Fierenz A Werfel L Rosin-Arbesfeld R Hofhuis J Thoms S. Systematic and quantitative analysis of stop codon readthrough in Rett syndrome nonsense mutations. J Mol Med. (2024) 102:641–53. doi: 10.1007/s00109-024-02436-6 38430393 PMC11055764

[72] Brendel C Belakhov V Werner H Wegener E Gärtner J Nudelman I . Readthrough of nonsense mutations in Rett syndrome: evaluation of novel aminoglycosides and generation of a new mouse model. J Mol Med. (2011) 89:389–98. doi: 10.1007/s00109-010-0704-4, 21120444 PMC3055984

[73] Merritt JK Collins BE Erickson KR Dong H Neul JL. Pharmacological read-through of R294x Mecp2 in a novel mouse model of Rett syndrome. Hum Mol Genet. (2020) 29:2461–70. doi: 10.1093/hmg/ddaa102, 32469049 PMC7471501

[74] Grimm NB Lee JT. Selective xi reactivation and alternative methods to restore Mecp2 function in Rett syndrome. Trends Genet. (2022) 38:920–43. doi: 10.1016/j.tig.2022.01.007, 35248405 PMC9915138

[75] Carrette LLG Wang CY Wei C Press W Ma W Kelleher RJ . A mixed modality approach towards xi reactivation for Rett syndrome and other X-linked disorders. Proc Natl Acad Sci USA. (2018) 115:E668–e75. doi: 10.1073/pnas.1715124115, 29282321 PMC5789928

[76] Carrette LLG Blum R Ma W Kelleher RJ Lee JT. Tsix-Mecp2 female mouse model for Rett syndrome reveals that low-level Mecp2 expression extends life and improves neuromotor function. Proc Natl Acad Sci USA. (2018) 115:8185–90. doi: 10.1073/pnas.1800931115, 30038001 PMC6094149

[77] Bhatnagar S Zhu X Ou J Lin L Chamberlain L Zhu LJ . Genetic and pharmacological reactivation of the mammalian inactive X chromosome. Proc Natl Acad Sci USA. (2014) 111:12591–8. doi: 10.1073/pnas.1413620111, 25136103 PMC4156765

[78] Przanowski P Wasko U Zheng Z Yu J Sherman R Zhu LJ . Pharmacological reactivation of inactive X-linked Mecp2 in cerebral cortical neurons of living mice. Proc Natl Acad Sci USA. (2018) 115:7991–6. doi: 10.1073/pnas.1803792115, 30012595 PMC6077728

[79] Sripathy S Leko V Adrianse RL Loe T Foss EJ Dalrymple E . Screen for reactivation of Mecp2 on the inactive X chromosome identifies the bmp/Tgf-Β superfamily as a regulator of Xist expression. Proc Natl Acad Sci USA. (2017) 114:1619–24. doi: 10.1073/pnas.1621356114, 28143937 PMC5321041

[80] Lee HM Kuijer MB Ruiz Blanes N Clark EP Aita M Galiano Arjona L . A small-molecule screen reveals novel modulators of Mecp2 and X-chromosome inactivation maintenance. J Neurodev Disord. (2020) 12:29. doi: 10.1186/s11689-020-09332-3, 33172406 PMC7657357

[81] Lou S R DJT Wasko UN Equbal Z Venkatesan S Braczyk K . Targeting microrna-dependent control of X chromosome inactivation improves the Rett syndrome phenotype. Nat Commun. (2025) 16:6169. doi: 10.1038/s41467-025-61092-7 40615387 PMC12227778

[82] Qian J Guan X Xie B Xu C Niu J Tang X . Multiplex epigenome editing of Mecp2 to rescue Rett syndrome neurons. Sci Transl Med. (2023) 15:eadd4666. doi: 10.1126/scitranslmed.add4666, 36652535 PMC11975455

[83] Wang J Wegener JE Huang TW Sripathy S De Jesus-Cortes H Xu P . Wild-type microglia do not reverse pathology in mouse models of Rett syndrome. Nature. (2015) 521:E1–4. doi: 10.1038/nature14444, 25993969 PMC4684952

[84] Akaba Y Shiohama T Komaki Y Seki F Ortug A Sawada D . Comprehensive volumetric analysis of Mecp2-null mouse model for Rett syndrome by T2-weighted 3d magnetic resonance imaging. Front Neurosci. (2022) 16:885335. doi: 10.3389/fnins.2022.885335, 35620663 PMC9127869

[85] Achilly NP He LJ Kim OA Ohmae S Wojaczynski GJ Lin T . Deleting Mecp2 from the cerebellum rather than its neuronal subtypes causes a delay in motor learning in mice. eLife. (2021) 10:4833. doi: 10.7554/eLife.64833, 33494858 PMC7837679

[86] Gagliardi A Giuliano E Venkateswararao E Fresta M Bulotta S Awasthi V . Biodegradable polymeric nanoparticles for drug delivery to solid tumors. Front Pharmacol. (2021) 12:601626. doi: 10.3389/fphar.2021.601626, 33613290 PMC7887387

[87] Vaughan HJ Green JJ Tzeng SY. Cancer-targeting nanoparticles for combinatorial nucleic acid delivery. Adv Mater. (2020) 32:1901081. doi: 10.1002/adma.201901081, 31222852 PMC6923623

[88] Sheinerman K Djukic A Tsivinsky VG Umansky SR. Brain-enriched micrornas circulating in plasma as novel biomarkers for Rett syndrome. PLoS One. (2019) 14:e0218623. doi: 10.1371/journal.pone.0218623, 31291284 PMC6619658

[89] Saby JN Peters SU Roberts TPL Nelson CA Marsh ED. Evoked potentials and EEG analysis in Rett syndrome and related developmental encephalopathies: towards a biomarker for translational research. Front Integr Neurosci. (2020) 14:30. doi: 10.3389/fnint.2020.00030, 32547374 PMC7271894

[90] Saby JN Mulcahey PJ Benke TA Peters SU Standridge SM Lieberman DN . Electroencephalographic correlates of clinical severity in the natural history study of Rtt and related disorders. Ann Neurol. (2024) 96:175–86. doi: 10.1002/ana.26948, 38721759 PMC12045323

[91] Saby JN Peters SU Benke TA Standridge SM Swanson LC Lieberman DN . Comparison of evoked potentials across four related developmental encephalopathies. J Neurodev Disord. (2023) 15:10. doi: 10.1186/s11689-023-09479-9, 36870948 PMC9985257

[92] Saby JN Marsh ED. Considerations and procedures for acquiring EEG as part of multi-site studies for Rett syndrome and other genetic neurodevelopmental disorders. Front Integr Neurosci. (2025) 19:1574758. doi: 10.3389/fnint.2025.1574758, 40552096 PMC12183233

[93] Saby JN Benke TA Peters SU Standridge SM Matsuzaki J Cutri-French C . Multisite study of evoked potentials in Rett syndrome. Ann Neurol. (2021) 89:790–802. doi: 10.1002/ana.26029, 33480039 PMC8882338

[94] Sysoeva OV Molholm S Djukic A Frey HP Foxe JJ. Atypical processing of tones and phonemes in Rett syndrome as biomarkers of disease progression. Transl Psychiatry. (2020) 10:188. doi: 10.1038/s41398-020-00877-4, 32522978 PMC7287060

[95] Palmieri M Pozzer D Landsberger N. Advanced genetic therapies for the treatment of Rett syndrome: state of the art and future perspectives. Front Neurosci. (2023) 17:1172805. doi: 10.3389/fnins.2023.1172805 37304036 PMC10248472

