# Correction: Disease-modifying therapies for Rett syndrome: a review for neurologists

**DOI:** 10.3389/fneur.2026.1828621

**Published:** 2026-04-20

**Authors:** Debopam Samanta

**Affiliations:** Division of Child Neurology, Department of Pediatrics, University of Arkansas for Medical Sciences, Little Rock, AR, United States

**Keywords:** AAV, gene therapy, neurodevelopmental disorders, pediatric neurology, precision therapeutics

In the published article, the reference “Sadhu C, Lyons C, Oh J, Jagadeeswaran I, Gray SJ, Sinnett SE. The efficacy of a human-ready mini MECP2 gene therapy in a pre-clinical model of Rett syndrome. *Genes*. (2023) 15:31. doi: 10.3390/genes15010031” was not cited in the article.

The citation has now been inserted in the Section 4.1.1.1, Paragraph Number 2 after first sentence following citation 52 and should read: “Preclinical validation of miniMECP2 was conducted by delivering scAAV-miniMECP2 directly into the brains of neonatal Mecp2-null mice (52, 53).”

A correction is also required in the caption of Figure 1C. It was previously published as “(C) Preclinical efficacy: In MECP2-null mice, scAAV-miniMECP2 improved neurological function, breathing, dendritic spine density, and survival without overexpression toxicity. miRARE restricted expression in wild-type neurons (~8%) but expanded it in deficient neurons (~40%), providing therapeutic benefit.”

The corrected caption of Figure 1C appears below.

“**(C)** Preclinical efficacy: In MECP2-null mice, scAAV-miniMECP2 improved neurological function, breathing, and survival without overexpression toxicity.”

In addition, a correction has been made to the Section 4.1.1.1 paragraph 2:

“Treated animals demonstrated significant improvements in neurological function, normalized breathing patterns, extended survival (from ~10 weeks to >1 year), and partial restoration of dendritic spine density-all without the toxicity associated with full-length MECP2 overexpression in wild-type mice.”

The corrected sentence appears below:

“Treated animals demonstrated significant improvements in neurological function, normalized breathing patterns, and extended survival without the toxicity associated with full-length MECP2 overexpression in wild-type mice.”

The original version of this article has been updated.
